# Assessment of Local Recurrence and Distant Metastasis After Curative Resection of Rectal Tumors: A Retrospective Study

**DOI:** 10.7759/cureus.107475

**Published:** 2026-04-21

**Authors:** Fahad Yasin, Sayed M Kazim, Hafsa Atiq, Abdullah Maqbool, Saqib Shakeel, Muhammad Awais, Fatima Tu Zahara, Ali Abaid, Shahid Khattak, Aamir A Syed

**Affiliations:** 1 Surgical Oncology, Sharif Medical and Dental College, Lahore, PAK; 2 Surgical Oncology, Shaukat Khanum Memorial Cancer Hospital and Research Centre, Lahore, PAK; 3 Internal Medicine, Arif Memorial Teaching Hospital, Lahore, PAK; 4 Surgery, Shalamar Medical and Dental College, Lahore, PAK; 5 Surgery, Hayyat Memorial Teaching Hospital, Lahore, PAK

**Keywords:** colorectal surgery, curative resection, distant metastasis, local recurrence, lymph node involvement, prognostic factors, rectal cancer

## Abstract

Background

Rectal cancer remains a significant global health burden, with rising incidence in younger populations and substantial risk of recurrence despite curative-intent resection. This study aimed to assess rates of local recurrence and distant metastasis following curative resection of rectal tumors and identify clinical and pathological predictors of oncologic outcomes.

Methods

This single-center retrospective study analyzed 289 patients who underwent curative resection for non-metastatic rectal adenocarcinoma at Shaukat Khanum Memorial Cancer Hospital and Research Center, Lahore, between 2013 and 2017. Demographic, clinicopathological, and treatment data were collected from medical records. Patients were followed for a median of 60 months according to a standardized surveillance protocol. Kaplan-Meier survival analysis and multivariate Cox proportional hazards regression were performed to identify predictors of local recurrence and distant metastasis.

Results

The cohort had a mean age of 44.3 ± 14.8 years with a male predominance (71.6%). Neoadjuvant chemoradiotherapy was administered to 78.9% of patients, and 55.4% received adjuvant chemotherapy. Local recurrence occurred in 17.6% (n=51) and distant metastasis in 22.8% (n=66), with lung (5.2%) and liver (4.5%) being the most common metastatic sites. The one-year, three-year, and five-year recurrence-free survival rates were 83.5% (95% CI: 79.2-87.8%), 66.6% (95% CI: 60.9-72.3%), and 52.8% (95% CI: 46.5-59.1%), respectively, with a mean recurrence-free survival of 76.3 months (95% CI: 71.05-81.08 months).

On multivariate analysis, independent predictors of local recurrence included signet ring cell histology (HR 3.42, 95% CI 1.98-5.91; p<0.001), ypN2 nodal status (HR 2.85, 95% CI 1.62-5.01; p<0.001), positive circumferential resection margin (HR 2.14, 95% CI 1.28-3.58; p=0.004), poor tumor differentiation (HR 1.96, 95% CI 1.18-3.26; p=0.009), and extra-levator abdominoperineal resection (HR 3.67, 95% CI 1.95-6.91; p<0.001). Predictors of distant metastasis included ypN2 status (HR 3.21, 95% CI 1.88-5.48; p<0.001), signet ring histology (HR 2.76, 95% CI 1.61-4.73; p<0.001), and positive circumferential resection margin (CRM) (HR 1.98, 95% CI 1.19-3.29; p=0.008).

Conclusions

Signet ring cell histology, advanced nodal disease (ypN2), positive circumferential resection margin, poor tumor differentiation, and low rectal tumors requiring extralevator abdominoperineal resection (ELAPR) are independent predictors of recurrence following curative resection of rectal cancer. These findings support risk-stratified postoperative surveillance and individualized adjuvant therapy strategies to improve long-term outcomes, particularly in high-risk patient populations.

## Introduction

Rectal cancer accounts for approximately 732,000 new cases globally in 2020, and its incidence is expected to rise to over 1.16 million by 2040 [1]. Advances in screening and treatment have significantly improved survival, with contemporary five-year relative survival rates for rectal cancer approaching 67% (ranging from 66.5-67% in recent estimates), compared to approximately 48% in the mid-1970s [2]. Despite these gains, five-year local recurrence and distant metastasis rates remain significant at approximately 7.3% and 22.6%, respectively, following curative resection for stage I-III rectal cancer [3].

The overall incidence among older adults (aged 50-64) has declined by 1.5% and 4.3% in those over 65 years, while rectal cancer incidence in individuals under 50 has increased by 1.8% annually [4]. Furthermore, the distribution of tumor sites exhibits significant diversity between age groups, and the median age of diagnosis is notably young (63 years for men and 65 years for women) [4]. As people age, the male-to-female incidence rate ratio likewise increases, rising from 1.10 in those aged 0-49 to 1.29 in those over 80 [4].

The stage-specific survival rates between colon and rectal cancers are comparable; the overall five-year survival rate for rectal cancer is approximately 67%, which is marginally better than that for colon cancer (64.2%) [2]. The mortality rate in men is 30-40% higher than in women, with possible reasons including differences in tumor biology or healthcare access [2].

Timely detection and early diagnosis remain key. For initial evaluation of rectal malignancies, flexible sigmoidoscopy is effective, but to detect synchronous lesions (observed in up to 4% of patients) elsewhere in the colon, colonoscopy is essential [5]. Contemporary rectal cancer management follows a multimodal paradigm that integrates surgery with systemic and radiation therapies. For locally advanced rectal cancer (LARC), the current standard of care includes neoadjuvant chemoradiotherapy (nCRT) followed by total mesorectal excision (TME), which significantly reduces local recurrence rates compared to surgery alone [6]. More recently, total neoadjuvant therapy (TNT) - delivering all chemotherapy and radiation preoperatively - has emerged as an alternative strategy that may improve pathological complete response rates and potentially facilitate organ preservation in selected patients [7]. Adjuvant chemotherapy is typically recommended based on final pathological staging, particularly for node-positive disease. This integrated, stage-adapted approach combining surgery, radiation oncology, and medical oncology represents the foundation of modern rectal cancer care [2, 6].

Total mesorectal excision (TME) offers superior local control and long-term survival compared to the older practice of blunt dissection and has become the standard surgical technique for rectal cancer [6, 8]. For early-stage distal tumors lacking aggressive features, local excision can be performed using transanal, transsphincteric, or transsacral approaches. However, recurrence remains a concern, with reported rates ranging from 7-21% for T1 tumors, warranting regular postoperative surveillance [9].

Despite the evolution in surgical techniques, even after curative resection, recurrence (local or distant) continues to affect a substantial subset of rectal cancer patients. This emphasizes the importance of understanding the risk factors associated with recurrence to optimize postoperative management and improve long-term outcomes and survival [3, 9].

Study objectives

The primary aim of this retrospective study was to assess the rates and timing of local recurrence and distant metastasis following curative resection of rectal tumors. Secondary objectives included identifying clinical and pathological predictors of recurrence, including tumor histology, differentiation, lymph node status, and receipt of adjuvant therapy; characterizing patterns of metastatic spread; and estimating recurrence-free survival at one, three, and five years.

By analyzing a five-year cohort of patients treated at Shaukat Khanum Memorial Cancer Hospital, this study provides outcome data from a high-volume tertiary cancer center in a resource-limited country. These insights will contribute to refining postoperative risk stratification and surveillance strategies, thereby improving long-term outcomes for rectal cancer patients.

## Materials and methods

Study design

This single-center retrospective study was conducted at Shaukat Khanum Memorial Cancer Hospital and Research Center, Lahore. Data were analyzed for rectal cancer patients treated between 2013 and 2017. Ethical approval was obtained from the Institutional Review Board of the hospital. Eligible patients were aged 18 years or older, with histopathologically confirmed, non-metastatic rectal adenocarcinoma who underwent curative resection. Exclusion criteria included metastatic disease at diagnosis, incomplete clinical or pathological records, and non-rectal gastrointestinal malignancies. Tumor staging was performed using the American Joint Committee on Cancer (AJCC) TNM staging system, Eighth edition.

Data collection

Demographic variables (age, sex, nationality), tumor characteristics (histological type, grade, size, location, lymph node status), and treatment details (surgical approach, postoperative complications, and adjuvant therapy) were collected from medical records. Patients were followed for a median of 60 months. Follow-up assessments were conducted in the outpatient department at three- to six-month intervals during the first two years and every 6-12 months until the fifth year. The follow-up protocol included physical examination, serum carcinoembryonic antigen (CEA) testing, imaging with CT or MRI when clinically indicated, and surveillance colonoscopy (at one year postoperatively, then every three to five years). Patient confidentiality was maintained throughout.

Surgical technique

All resections were performed with curative intent, adhering to the principle of total mesorectal excision (TME). The choice of procedure (low anterior resection, ultra-low anterior resection, or abdominoperineal resection) was individualized based on tumor stage, location, and pelvic anatomy. Intersphincteric resections were not performed in this cohort. Lateral lymph node dissection was not routinely performed. Indocyanine green (ICG) was not utilized for anastomotic perfusion assessment or lymph node mapping in this study. Local excision techniques (transanal, transsphincteric, or transsacral) were reserved for early-stage distal tumors without aggressive histological features.

Statistical analysis

Descriptive statistics were used to summarize baseline patient and tumor characteristics. Kaplan-Meier survival analysis was employed to estimate recurrence-free survival (RFS) and overall survival (OS), with survival curves compared using the log-rank test. Survival probabilities with 95% confidence intervals were calculated at one, three, and five years.

Recurrence-free survival (RFS) was defined as the time from the date of surgery to the first documented event: local recurrence, distant metastasis, or death from any cause. Patients without an event at the last follow-up were censored at the date of last documented contact. Local recurrence was defined as histopathologically confirmed adenocarcinoma within the pelvis or unequivocal imaging evidence with compatible clinical findings. Distant metastasis was defined as histopathologically confirmed disease in distant organs or unequivocal imaging evidence with clinical progression.

The Cox proportional hazards regression model was applied for univariate and multivariate analysis to identify predictors of recurrence and distant metastasis. For multivariate models, variables with p<0.10 on univariate analysis were considered for inclusion, along with clinically important variables (age, sex), regardless of statistical significance. Final multivariate models were constructed using forward stepwise selection with entry criterion p<0.05 and removal criterion p>0.10. Separate multivariate models were developed for 1) local recurrence as the event of interest and 2) distant metastasis as the event of interest; patients experiencing the competing event were censored at that time. Distant metastasis was treated strictly as an outcome variable and was not included as a predictor variable in any survival analysis.

The proportional hazards assumption was tested using scaled Schoenfeld residuals and log-minus-log plots; no violations were detected. A p-value of <0.05 was considered statistically significant. Analyses were performed using Stata/IC 15.1 for Mac (StataCorp LLC, College Station, Texas). Survival outcomes were assessed over a five-year period.

## Results

Patient demographics and baseline characteristics

A total of 289 patients underwent curative-intent resection for rectal adenocarcinoma at Shaukat Khanum Memorial Cancer Hospital and Research Center between 2013 and 2017 and were included in the analysis. The mean age at diagnosis was 44.3 ± 14.8 years (Table 1). There was a marked male predominance (71.6%, n=207).

**Table 1:** Baseline demographic and clinicopathological characteristics *CRM status was unknown for 9.0% of patients as this detail was not documented in the original pathology reports CRM - circumferential resection margin

Characteristic	n=289 (%)
Age (years), mean ± SD	44.3 ± 14.8
Gender	
Male	207 (71.6)
Female	82 (28.4)
Neoadjuvant therapy	
Chemoradiotherapy	228 (78.9)
Chemotherapy alone	7 (2.4)
Radiotherapy alone	7 (2.4)
None	47 (16.3)
Tumor type	
Adenocarcinoma	211 (73.0)
Signet ring cell adenocarcinoma	62 (21.5)
Mucinous adenocarcinoma	9 (3.1)
Melanoma	6 (2.1)
Squamous cell carcinoma	1 (0.3)
Tumor differentiation	
Well differentiated	32 (11.1)
Moderately differentiated	187 (64.7)
Poorly differentiated	61 (21.1)
Unknown	9 (3.1)
Circumferential resection margin	
Negative	174 (60.2)
Positive	88 (30.4)
Unknown*	26 (9.0)
Lymph node yield (total harvested)	
<5 nodes	24 (8.3)
5-10 nodes	18 (6.2)
10-15 nodes	120 (41.5)
>15 nodes	127 (43.9)
ypN stage (post-neoadjuvant)	
ypN0	177 (61.2)
ypN1	86 (29.7)
ypN2	26 (9.0)
pT stage	
pT1	1 (0.3)
pT2	19 (6.6)
pT3	240 (83.0)
pT4	29 (10.0)
Surgical procedure	
Low anterior resection	92 (31.8)
Ultra-low anterior resection	78 (27.0)
Abdominoperineal resection	82 (28.4)
Extralevator abdominoperineal resection	37 (12.8)
Adjuvant chemotherapy	160 (55.4)
Local recurrence	51 (17.6)
Distant metastasis	66 (22.8)

All procedures were performed using a laparoscopic approach; no robotic resections were undertaken during the study period.

Treatment characteristics and follow-up

Neoadjuvant chemoradiotherapy was administered to 78.9% (n=228) of patients. Postoperatively, 55.4% (n=160) received adjuvant chemotherapy.

Patients were followed according to a standardized surveillance protocol. Clinical assessment, serum carcinoembryonic antigen (CEA) testing, and cross-sectional imaging (CT chest/abdomen and MRI pelvis) were performed at three- to six-month intervals during the first two years and annually thereafter up to five years. Surveillance colonoscopy was performed at one, three, and five years. The median follow-up duration was 60 months (range: 1-99 months).

Surgical procedures and tumor location

Low anterior resection (LAR) was the most frequently performed procedure (n=92), followed by abdominoperineal resection (APR) (n=82), extralevator abdominoperineal resection (ELAPR) (n=37), and ultra-low anterior resection (n=78).

The type of surgical procedure demonstrated a significant association with local recurrence (p<0.001). ELAPR was associated with the highest local recurrence rate (35.1%, 13/37), followed by APR (18.3%, 15/82). Sphincter-preserving procedures were associated with lower recurrence rates, including LAR (9.0%, 17/92) and ultra-low anterior resection (7.7%, 6/78).

Tumor location relative to the peritoneal reflection was also significantly associated with recurrence (p=0.042). Tumors located below the peritoneal reflection demonstrated higher recurrence rates (23.7%, 32/135) compared with tumors above the reflection (10.9%, 16/147).

Pathological characteristics

Conventional adenocarcinoma was the predominant histologic subtype (73.0%, n=211). Signet ring cell carcinoma accounted for 21.5% (n=62), mucinous adenocarcinoma for 3.1% (n=9), melanoma for 2.1% (n=6), and squamous cell carcinoma for 0.3% (n=1).

Most tumors were moderately differentiated (64.7%, n=187), while 21.1% (n=61) were poorly differentiated and 11.1% (n=32) were well differentiated.

Pathologic T stage revealed a predominance of locally advanced disease, with 83.0% (n=240) classified as pT3. Post-neoadjuvant nodal involvement (ypN+) was observed in 38.8% (n=112), including ypN1 disease in 29.7% (n=86) and ypN2 disease in 9.0% (n=26).

Circumferential resection margin (CRM) was positive in 30.4% (n=88), negative in 60.2% (n=174), and undocumented in 9.0% (n=26). Total lymph node yield was <5 in 8.3% (n=24), 5-10 in 6.2% (n=18), 10-15 in 41.5% (n=120), and >15 in 43.9% (n=127).

Patterns of recurrence and metastasis

During follow-up, local recurrence occurred in 17.6% (n=51) of patients.

Distant metastasis developed in 22.8% (n=66). The most common sites were lung (5.2%, n=15) and liver (4.5%, n=13), followed by peritoneum (1.4%, n=4) and bone (1.4%, n=4). Multi-organ metastases were observed in 8.0% (n=23).

Recurrence-free survival

Kaplan-Meier analysis was performed to estimate recurrence-free survival (RFS). The median RFS was not reached, as fewer than 50% of patients developed local recurrence during the follow-up period.

The estimated mean recurrence-free survival was 76.29 months (95% CI: 71.05-81.08 months), corresponding to approximately 6.4 years.

**Figure 1:**
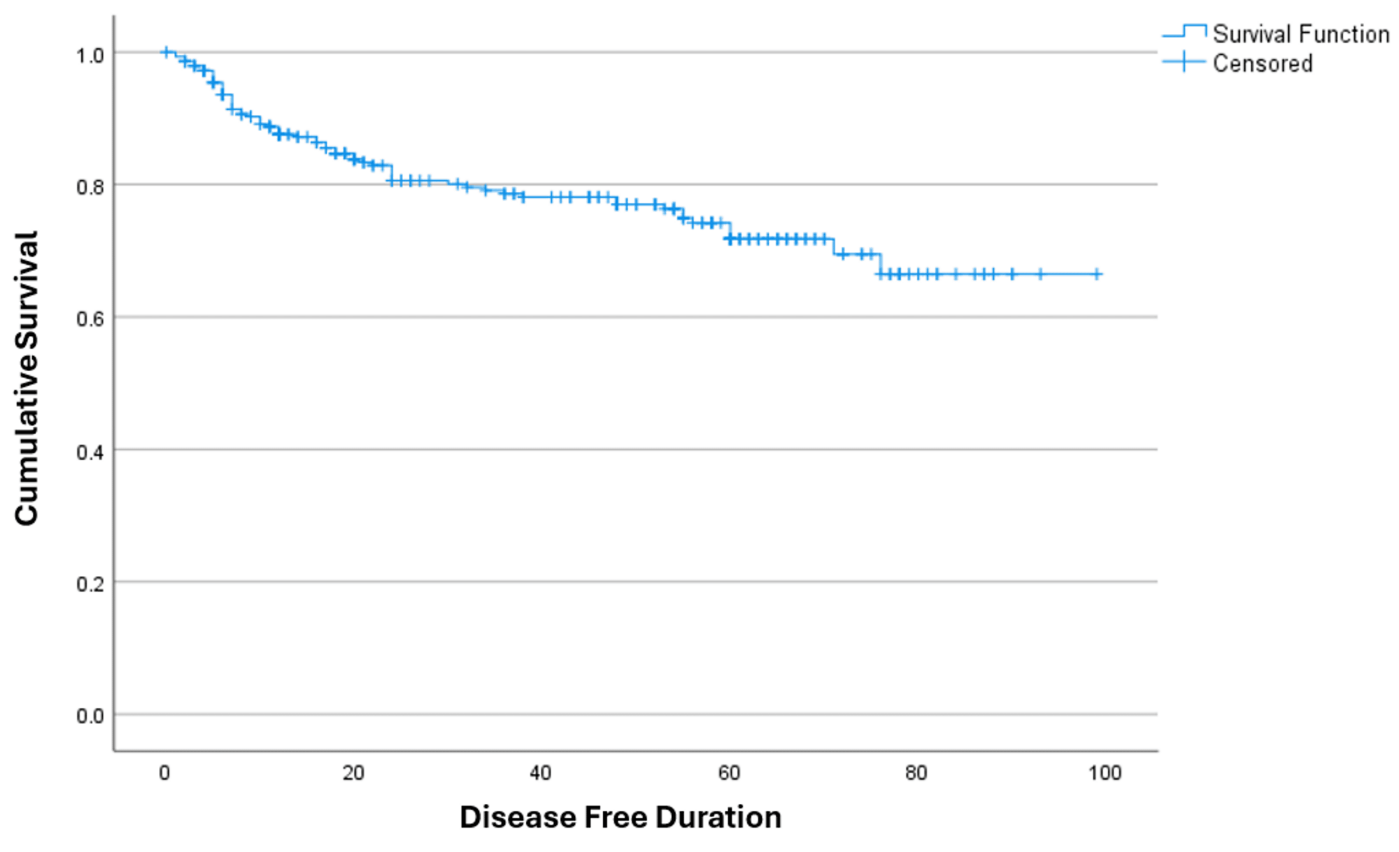
Kaplan-Meier curve following curative resection for rectal cancer The estimated mean survival was 76.29 months (95% CI: 71.05-81.08 months), indicating an average survival of approximately 6.4 years in the study population

The estimated one-year, three-year, and five-year recurrence-free survival rates were 83.5% (95% CI: 79.2-87.8%), 66.6% (95% CI: 60.9-72.3%), and 52.8% (95% CI: 46.5-59.1%), respectively (Figure 1). These findings reflect durable local disease control following curative-intent resection in the majority of patients.

Predictors of local recurrence

On multivariate Cox proportional hazards analysis (Table 2), signet ring cell histology was independently associated with a significantly increased risk of local recurrence compared with conventional adenocarcinoma (HR 3.42, 95% CI 1.98-5.91; p<0.001).

**Table 2:** Multivariate predictors of local recurrence CRM - circumferential resection margin; ELAPR - extralevator abdominoperineal resection; LAR - low anterior resection

Variable	Hazard ratio	95% CI	p-value
Signet ring histology (vs. adenocarcinoma)	3.42	1.98–5.91	<0.001
ypN2 (vs. ypN0)	2.85	1.62–5.01	<0.001
Positive CRM (vs. negative)	2.14	1.28–3.58	0.004
Poor differentiation (vs. moderate)	1.96	1.18–3.26	0.009
ELAPR (vs. LAR)	3.67	1.95–6.91	<0.001
Age	1.01	0.99–1.03	0.312
Gender (male vs. female)	1.12	0.68–1.85	0.652

Advanced nodal disease (ypN2) was a strong predictor of recurrence (HR 2.85, 95% CI 1.62-5.01; p<0.001). Positive CRM status independently increased the risk of recurrence (HR 2.14, 95% CI 1.28-3.58; p=0.004). Poor tumor differentiation was also associated with higher recurrence risk (HR 1.96, 95% CI 1.18-3.26; p=0.009).

The type of surgical procedure remained an independent predictor after adjustment for confounding factors. Compared with low anterior resection (reference), ELAPR was associated with the highest recurrence risk (HR 3.67, 95% CI 1.95-6.91; p<0.001).

Predictors of distant metastasis

In multivariate analysis evaluating predictors of distant metastasis (Table 3), ypN2 nodal status was the strongest independent predictor (HR 3.21, 95% CI 1.88-5.48; p<0.001).

Signet ring cell histology (HR 2.76, 95% CI 1.61-4.73; p<0.001) and positive CRM (HR 1.98, 95% CI 1.19-3.29; p=0.008) were also independently associated with increased risk of distant metastatic spread.

**Table 3:** Multivariate predictors of distant metastasis CRM - circumferential resection margin; ELAPR - extralevator abdominoperineal resection; LAR - low anterior resection

Variable	Hazard ratio	95% CI	p-value
ypN2 (vs. ypN0)	3.21	1.88–5.48	<0.001
Signet ring histology (vs. adenocarcinoma)	2.76	1.61–4.73	<0.001
Positive CRM (vs. negative)	1.98	1.19–3.29	0.008
Poor differentiation (vs. moderate)	1.72	1.04–2.85	0.034
ELAPR (vs. LAR)	2.14	1.18–3.88	0.012

## Discussion

In this large single‑institution cohort of rectal cancer patients treated with standardized multidisciplinary care, we identified several clinicopathological factors that significantly influence local recurrence and distant metastasis following curative‑intent resection. The observed local recurrence rate of 17.6% and distant metastasis rate of 22.8% highlight the ongoing challenge of durable disease control even in the era of neoadjuvant chemoradiotherapy.

Histological subtypes and prognosis

Our findings confirm that histologic subtype and nodal burden are among the strongest determinants of oncologic outcome. Signet ring cell histology was independently associated with a markedly increased risk of local recurrence (HR 3.42, 95% CI 1.98-5.91; p<0.001, Table 2) and distant metastasis (HR 2.76, 95% CI 1.61-4.73; p<0.001, Table 3). This aligns with a recent Swedish population-based study by Enblad et al., which demonstrated that signet ring cell (SRC) colorectal cancer patients had significantly worse five-year overall survival compared to non-SRC patients (18% vs. 32%, p=0.004) and a higher risk of open-close laparotomy during cytoreductive surgery (22% vs. 13%, p=0.04) [10]. The aggressive behavior of signet ring tumors is further elucidated by An et al., who performed comprehensive genomic and immune profiling of colorectal SRC and found that these tumors exhibit a distinct molecular landscape with fewer mutations in conventional driver genes (APC 32% vs. 74%, KRAS 16% vs. 42%) but higher frequencies of alterations in SMAD4, RNF43, and MYC [11]. Notably, SRC demonstrates a unique "pseudo-T cell-inflamed" tumor microenvironment with increased total CD8+ tumor-infiltrating lymphocytes but reduced PD-1+CD8+ TIL infiltration, which may contribute to treatment resistance and poor prognosis [11]. Mucinous adenocarcinoma, while less common in our cohort (3.1%), has been associated with microsatellite stability and adverse biological features in large population-based studies [12]. These observations reinforce the necessity of incorporating specific tumor histology into clinical decision-making and risk stratification protocols.

Lymph node involvement as a prognostic indicator

Lymph node involvement proved to be another crucial prognostic indicator in our analysis. Post‑neoadjuvant ypN2 nodal status emerged as a powerful predictor of both local recurrence (HR 2.85, 95% CI 1.62-5.01; p<0.001, Table 2) and distant metastasis (HR 3.21, 95% CI 1.88-5.48; p<0.001, Table 3). This finding is consistent with Nakamura et al., who reported that in patients receiving neoadjuvant chemoradiotherapy for rectal cancer, ypN2 was the only independent prognostic factor for both disease-free survival (p=0.0019) and overall survival (p=0.0064) on multivariate analysis [13]. Similarly, a study by the same group demonstrated that among 105 patients with locally advanced rectal cancer, ypN2 was independently associated with significantly worse recurrence-free survival (p=0.0007) and overall survival (p=0.0012) [14]. Residual nodal disease after neoadjuvant therapy reflects persistent biologically aggressive disease and should be considered a key indicator for intensified adjuvant therapy consideration [13, 14].

Circumferential resection margin and quality of surgery

Circumferential resection margin (CRM) positivity was independently associated with adverse outcomes in our cohort (HR 2.14 for local recurrence, HR 1.98 for distant metastasis). The clinical significance of CRM involvement has been extensively characterized in a nationwide Dutch population-based study by Hugen et al., which analyzed 3020 patients with stage III rectal cancer [15]. This study demonstrated that the mode of CRM involvement determines its impact on outcomes: CRM involvement by primary tumor invasion and multiple factors were associated with poor overall survival, whereas CRM involvement by lymph node metastasis alone did not carry the same prognostic weight [15]. The rates of local recurrence and distant metastasis were significantly related to the nature of CRM involvement, with the highest rates observed when multiple factors contributed to margin positivity [15]. These findings underscore the importance of detailed pathological reporting of CRM status and the need for meticulous surgical technique to achieve clear margins [15, 16].

Surgical approach and recurrence risk

The association between the type of surgical procedure and recurrence in our cohort likely reflects both technical and tumor‑biological factors. Extralevator abdominoperineal resection (ELAPR), typically indicated for very low or fixed tumors, had the highest recurrence hazard (HR 3.67, 95% CI 1.95-6.91; p<0.001). As reviewed by Wilkins et al., APR variants have historically been associated with higher rates of intraoperative perforation and CRM involvement compared to anterior resection, contributing to increased local recurrence risk [16]. However, these authors caution that patient selection and tumor biology play significant roles, as tumors requiring APR are inherently more advanced and anatomically challenging [16]. The introduction of ELAPE aimed to reduce CRM positivity by creating a cylindrical specimen, yet studies have not consistently demonstrated superior oncological outcomes, and wound complication rates may be higher [16]. These considerations emphasize that surgical technique must be tailored to individual patient anatomy and tumor characteristics.

Tumor location and recurrence patterns

The biological interplay between tumor location and patterns of metastasis is complex. Tumors located below the peritoneal reflection demonstrated significantly higher recurrence rates (23.7% vs. 10.9%, p=0.042) in our cohort. Low‑lying rectal tumors have distinct lymphovascular drainage pathways, including potential involvement of lateral pelvic lymph nodes, which can influence patterns of systemic spread. A recent MRI-based scoring system developed by Cho et al. for predicting lateral local recurrence in locally advanced low rectal cancer incorporated pretreatment lateral lymph node size and extramural venous invasion (EMVI), achieving excellent predictive performance (area under the curve 0.90-0.92) [17]. Their study of 607 patients demonstrated that selective lateral lymph node dissection based on these imaging features may reduce lateral recurrence while avoiding unnecessary extended lymphadenectomy [17]. The distal internal iliac compartment was identified as having the highest frequency of residual lymph nodes after neoadjuvant therapy, suggesting this region warrants particular attention during surgical planning [17].

Recurrence surveillance and follow-up protocol

Patients in our cohort underwent standardized postoperative surveillance according to a rigorous institutional protocol: clinical examination, serum carcinoembryonic antigen (CEA) measurement, and cross-sectional imaging (CT chest/abdomen and MRI pelvis) at three- to six-month intervals for the first two years, and annually thereafter through the fifth year. Surveillance colonoscopy was performed at one, three, and five years postoperatively. This protocol aligns closely with current international guideline recommendations summarized in a comprehensive umbrella review by Negoi, which synthesized evidence from existing guidelines regarding optimal follow-up strategies [18]. The review emphasizes that intensive surveillance during the first two to three years is justified as the majority of recurrences occur during this period, and that CEA monitoring combined with regular imaging improves detection of potentially curable recurrences [18]. Current guidelines from NCCN and ESMO similarly advocate for risk-stratified surveillance protocols, with high-risk patients potentially benefiting from more frequent imaging [18, 19].

Technical advances in lymph node assessment

Regarding lymph node harvest, emerging evidence supports the use of indocyanine green (ICG) fluorescence guidance to optimize lymph node retrieval and improve surgical precision. A systematic review by Kehagias et al. demonstrated that ICG-guided lateral lymph node dissection is associated with a significantly increased number of harvested lymph nodes, reduced blood loss, and decreased operative time compared to conventional dissection [20]. More recently, Pacilli et al. reported that preoperative peritumoral and intraoperative intravenous ICG injection in minimally invasive rectal cancer surgery was significantly associated with increased lymph node yield (β=3.65, p=0.002) and a higher number of positive nodes (β=0.85, p=0.028), indicating improved oncologic accuracy [21]. Notably, no anastomotic leaks occurred in the ICG group compared to a 10% leak rate in the control group, suggesting potential benefits for anastomotic perfusion assessment [21]. While our study did not utilize ICG, this technology represents an important advancement for centers performing extended lymphadenectomies, particularly in the setting of suspected lateral pelvic lymph node involvement.

Adjuvant therapy considerations

In our cohort, 55.4% of patients received adjuvant chemotherapy following resection. The recurrence rate in the adjuvant chemotherapy group was 18.1% compared to 16.8% in those who did not receive adjuvant therapy (p=0.78). This non-significant difference likely reflects confounding by indication, as patients with higher-risk pathological features (node-positive disease, poor differentiation, positive CRM) were preferentially selected for adjuvant treatment. Contemporary guidelines acknowledge this complexity: for patients who have received neoadjuvant therapy, adjuvant chemotherapy decisions are increasingly individualized based on ypStage and tumor regression grade [18]. The ADORE trial and subsequent meta-analyses have demonstrated that patients with ypStage III or poor response to neoadjuvant therapy may derive survival benefit from oxaliplatin-based adjuvant regimens [22]. With the increasing adoption of total neoadjuvant therapy (TNT), which delivers all chemotherapy prior to surgery, the role of postoperative adjuvant therapy is evolving and should be determined by multidisciplinary teams based on comprehensive risk assessment [18, 19].

Biological mechanisms of recurrence

Understanding the biological mechanisms underlying recurrence is essential for developing targeted therapeutic strategies. The distinct molecular profile of signet ring cell carcinoma identified by An et al. provides insights into its aggressive behavior: lower mutation rates in APC and KRAS but enrichment in SMAD4, RNF43, and MYC alterations, coupled with a unique immune microenvironment characterized by increased CD8+ TILs but reduced PD-1+CD8+ TIL infiltration [11]. Interestingly, high PD-1+CD8+ TILs in intratumoral regions significantly predicted longer disease-free and overall survival in SRC patients, suggesting potential for immunotherapy in selected cases [11]. For conventional adenocarcinoma, persistent nodal disease after neoadjuvant therapy (ypN+) likely reflects intrinsic chemoradioresistance, which may be mediated by cancer stem cell populations, DNA repair pathway alterations, or tumor microenvironment factors [13, 14]. Future research incorporating molecular biomarkers, circulating tumor DNA monitoring, and advanced imaging parameters may enable earlier detection of molecular relapse and more precise targeting of adjuvant therapy [23].

Comparison with national and international data

Our cohort demonstrated a mean age of 44.3 years, notably younger than the median age reported in Western populations (63-65 years) [1, 2]. This younger age at presentation is consistent with the increasing incidence of early-onset colorectal cancer observed globally, particularly in developing countries [2]. The proportion of signet ring cell carcinoma in our cohort (21.5%) is substantially higher than the 1.7% reported in the Surveillance, Epidemiology, and End Results (SEER) database for young adults [12], suggesting either a true epidemiological difference or referral bias to our tertiary center. The local recurrence rate (17.6%) and distant metastasis rate (22.8%) in our cohort are comparable to contemporary international series, though direct comparison is limited by variations in neoadjuvant therapy utilization and follow-up protocols. Our five-year recurrence-free survival (52.8%) reflects the advanced stage at presentation (83% pT3, 38.8% node-positive), consistent with real-world data from high-volume centers in developing nations. A Japanese series by Nakamura et al. reported five-year recurrence-free survival of 79% and overall survival of 80% in patients receiving neoadjuvant chemoradiotherapy, with similar prognostic factors including ypN2 status [13].

Limitations

This study has several limitations that warrant acknowledgment. First, the retrospective, single-center design may introduce selection bias and limit the generalizability of findings. Second, despite comprehensive data collection, we were unable to assess several important confounding factors that influence survival, including:

Extramural vascular invasion (EMVI), which has been identified as an independent predictor of lymph node metastasis and recurrence, was incorporated into predictive models for lateral lymph node dissection by Cho et al. [17]. Lateral pelvic lymph node status, which is particularly relevant for distal rectal tumors and may require dedicated imaging or selective dissection based on MRI criteria [17]. Lymphovascular invasion and perineural invasion are both established risk factors for nodal metastasis and poor outcomes. Tumor regression grade (TRG) following neoadjuvant therapy, which provides important prognostic information beyond ypStage [14]. Molecular markers, such as microsatellite instability, RAS/BRAF mutation status, and immune cell infiltration patterns, have demonstrated prognostic significance in recent studies [11].

Third, the absence of a control group precludes definitive conclusions about the comparative effectiveness of different treatment strategies. Fourth, variations in treatment protocols over the study period (2013-2017) may have influenced outcomes, particularly with the evolution of total neoadjuvant therapy approaches. Finally, while our sample size of 289 patients is substantial, it may limit statistical power for subgroup analyses and the detection of smaller effect sizes.

These limitations may have resulted in underestimation or overestimation of certain risk factors' true prognostic impact. For instance, the non-significant finding for adjuvant therapy may reflect unmeasured confounding rather than a true lack of efficacy. Similarly, the absence of EMVI and lateral node data means our risk models are incomplete, potentially missing important predictors that could refine patient selection for intensified surveillance or additional therapy.

Despite these limitations, the study's strengths include its large sample size, standardized treatment protocols, rigorous long-term follow-up with a median of 60 months, and comprehensive multivariate analysis identifying key prognostic factors.

Implications for clinical practice and future research

Despite these limitations, our findings have several important clinical implications. First, patients with signet ring cell histology, ypN2 disease, or positive CRM represent a high-risk population that may benefit from intensified surveillance protocols, including more frequent imaging and consideration of extended adjuvant therapy. The distinct molecular and immune profile of SRC suggests that these patients may also be candidates for clinical trials investigating novel therapeutic approaches, including immunotherapy for the subset with favorable immune infiltration patterns [11]. Second, tumor location and surgical procedure type should inform recurrence risk counseling and follow-up planning, with low rectal tumors requiring ELAPR warranting particular attention. Third, our data support the routine inclusion of detailed histopathological features in multidisciplinary team discussions to guide personalized treatment decisions, consistent with current guideline recommendations [18, 19].

Future prospective, multi-center studies should address the limitations identified herein. Specifically, such studies should incorporate comprehensive pathological assessment including EMVI, perineural invasion (PNI), lymphovascular invasion (LVI), and tumor regression grade (TRG) using standardized reporting protocols; utilize standardized preoperative imaging protocols to evaluate lateral pelvic lymph nodes, incorporating validated scoring systems [17]; collect biospecimens for molecular profiling (MSI status, gene mutations, immune infiltrate) to enable integration of biomarkers into risk stratification; employ standardized treatment protocols aligned with contemporary guidelines, including consideration of total neoadjuvant therapy for appropriate patients; include centralized pathology review to ensure consistent classification of histological subtypes and margin status; and utilize propensity score matching or instrumental variable analysis to better control for confounding by indication in treatment effect estimation.

The integration of circulating tumor DNA (ctDNA) monitoring into surveillance protocols represents a promising avenue for early detection of molecular relapse before clinical manifestation, as highlighted in recent guideline reviews [18, 23]. Similarly, artificial intelligence-based risk prediction models incorporating clinical, pathological, and imaging data may enable truly personalized surveillance intensity and adjuvant therapy selection [18]. Collectively, these advances, combined with the prognostic factors identified in our study, hold promise for improving outcomes in this challenging disease.

## Conclusions

In this cohort of rectal cancer patients undergoing curative-intent resection, signet ring cell histology, ypN2 nodal status, positive circumferential resection margin, poor tumor differentiation, and low rectal tumors requiring ELAPR were identified as independent predictors of local recurrence and distant metastasis. Overall, local recurrence-free survival was favorable, with a mean RFS of 76.3 months, but patients with high-risk pathological features remain at significantly elevated risk of recurrence.

These findings underscore the importance of accurate risk stratification, meticulous surgical technique, and personalized postoperative management, including consideration of intensified adjuvant therapy and vigilant follow-up for high-risk patients. Prospective multi-center studies are warranted to validate these findings and further refine risk stratification models. Incorporating both clinical and pathological risk factors into treatment planning may help optimize long-term oncologic outcomes in rectal cancer.

## References

[1] Morgan E, Arnold M, Gini A (2023). Global burden of colorectal cancer in 2020 and 2040: incidence and mortality estimates from GLOBOCAN. Gut.

[2] Maqbool S, Khan I, Rehman A (2025). Demographic and clinical disparities in rectal cancer survival: a retrospective cohort analysis using Surveillance, Epidemiology, and End Results (SEER) data (2014-2020). J Clin Oncol.

[3] Qaderi SM, Galjart B, Verhoef C (2021). Disease recurrence after colorectal cancer surgery in the modern era: a population-based study. Int J Colorectal Dis.

[4] Bailey CE, Hu CY, You YN (2015). Increasing disparities in the age-related incidences of colon and rectal cancers in the United States, 1975-2010. JAMA Surg.

[5] (2025). Colorectal Cancer Screening. MSD Manual Professional Edition. Reviewed/Revised May.

[6] Serra-Aracil X, Pericay C, Cidoncha A (2025). Chemoradiotherapy and local excision vs total mesorectal excision in T2-T3ab, N0, M0 rectal cancer: the TAUTEM randomized clinical trial. JAMA Surg.

[7] Shchatsko A, Balvardi S, Brazelle M (2025). Oncologic outcomes of organ preservation in patients with rectal adenocarcinoma treated with total neoadjuvant therapy: a single-center study. J Clin Oncol.

[8] Aref A, Abdalla A, Bhullar JS, Knoll E (2024). Perspective on the PROSPECT: the conundrum of managing T3n0-N1 mid and upper rectal cancer. Dis Colon Rectum.

[9] (2025). Fundamentals of Rectal Cancer Surgery: Management of Local Recurrences. ASCRS U Fundamentals of Rectal Cancer Surgery. Updated.

[10] Enblad M, Ghanipour L, Palmer G, Valdimarsson V, Bexe Lindskog E, Cashin P (2025). Prognosis and clinical characteristics of signet ring cell colorectal peritoneal metastases - a Swedish population-based study. Eur J Surg Oncol.

[11] An Y, Zhou J, Su L (2026). Clinicopathologic, genetic and immune cell infiltration analysis of colorectal signet ring cell carcinoma with comparison to conventional adenocarcinoma. J Natl Cancer Cent.

[12] Hugen N, Verhoeven RH, Lemmens VE (2015). Colorectal signet-ring cell carcinoma: benefit from adjuvant chemotherapy but a poor prognostic factor. Int J Cancer.

[13] Nakamura T, Yamashita K, Sato T, Ema A, Naito M, Watanabe M (2014). Neoadjuvant chemoradiation therapy using concurrent S-1 and irinotecan in rectal cancer: impact on long-term clinical outcomes and prognostic factors. Int J Radiat Oncol Biol Phys.

[14] Nakamura T, Sato T, Hayakawa K, Koizumi W, Kumagai Y, Watanabe M (2019). Strategy to avoid local recurrence in patients with locally advanced rectal cancer. Radiat Oncol.

[15] Hugen N, Voorham QJ, Beets GL, Loughrey MB, Snaebjornsson P, Nagtegaal ID (2024). The mode of circumferential margin involvement in rectal cancer determines its impact on outcomes: a population-based study. Eur J Surg Oncol.

[16] Wilkins S, Yap R, Mendis S, Carne P, McMurrick PJ (2022). Surgical techniques for abdominoperineal resection for rectal cancer: one size does not fit all. Front Surg.

[17] Cho MJ, Han K, Shin HJ, Koom WS, Lee KY, Kim JH, Lim JS (2025). MRI-based scoring systems for selective lateral lymph node dissection in locally advanced low rectal cancer after neoadjuvant chemoradiotherapy. Eur Radiol.

[18] Negoi I (2025). Guidance for the management of rectal cancer: an umbrella review of existing guidelines regarding surgical anatomy, adjuvant therapy, follow-up and surveillance, specific considerations, documentation, and palliative care. Cureus.

[19] Benson AB, Venook AP, Al-Hawary MM (2022). Rectal Cancer, Version 2.2022, NCCN Clinical Practice Guidelines in Oncology. J Natl Compr Canc Netw.

[20] Kehagias D, Lampropoulos C, Bellou A, Kehagias I (2024). The use of indocyanine green for lateral lymph node dissection in rectal cancer-preliminary data from an emerging procedure: a systematic review of the literature. Tech Coloproctol.

[21] Pacilli M, Pavone G, Lamanna E, Picciariello A, De Fazio M, Ambrosi A, Tartaglia N (2025). Impact of preoperative indocyanine green injection on intraoperative decision-making and lymph node harvest in rectal cancer surgery. Front Surg.

[22] Rödel C, Graeven U, Fietkau R (2015). Oxaliplatin added to fluorouracil-based preoperative chemoradiotherapy and postoperative chemotherapy of locally advanced rectal cancer (the German CAO/ARO/AIO-04 study): final results of the multicentre, open-label, randomised, phase 3 trial. Lancet Oncol.

[23] Negoi I (2025). Personalized surveillance in colorectal cancer: Integrating circulating tumor DNA and artificial intelligence into post-treatment follow-up. World J Gastroenterol.

